# Induced clustering of *Escherichia coli* by acoustic fields

**DOI:** 10.1038/s41598-018-22960-z

**Published:** 2018-03-16

**Authors:** Salomé Gutiérrez-Ramos, Mauricio Hoyos, J. C. Ruiz-Suárez

**Affiliations:** 1 Laboratoire de Physique et Mécanique des Milieux Hétérogènes (PMMH UMR 7636) CNRS, ESPCI Paris, PSL Research University, Sorbonne Université, Université Paris Diderot, 10 rue Vauquelin, 75005 Paris, France; 2 Centro de Investigación y de Estudios Avanzados, Unidad Monterrey, PIIT Autopista al Aeropuerto Km. 9.5, Apodaca, Nuevo León, 66600 Mexico

## Abstract

Brownian or self-propelled particles in aqueous suspensions can be trapped by acoustic fields generated by piezoelectric transducers usually at frequencies in the megahertz. The obtained confinement allows the study of rich collective behaviours like clustering or spreading dynamics in microgravity-like conditions. The acoustic field induces the levitation of self-propelled particles and provides secondary lateral forces to capture them at nodal planes. Here, we give a step forward in the field of confined active matter, reporting levitation experiments of bacterial suspensions of *Escherichia coli*. Clustering of living bacteria is monitored as a function of time, where different behaviours are clearly distinguished. Upon the removal of the acoustic signal, bacteria rapidly spread, impelled by their own swimming. Nevertheless, long periods of confinement result in irreversible bacteria entanglements that could act as seeds for levitating bacterial aggregates.

## Introduction

Since the seminal work of T. Vicsek *et al*. in 1995 on self**-**ordered motion in inert systems^1^, research in active matter has become a well-established field engaging both physicists and biologists. The main characteristic of active matter is that it is constituted by self-driven units or active particles containing internal degrees of freedom, with the ability to convert stored or ambient free energy into movement. Active matter exhibits intriguing non-equilibrium properties, emergent structures with collective behaviours, bizarre fluctuations, unusual mechanical and rheological properties, wave propagation and sustained oscillations among others^2^. The confinement of active matter has helped to evaluate collective phenomena of some self-propelled entities, for example nano-rods^3^, Janus particles^4^, and colloidal rotors^5–7^, among others.

A fascinating case of a suspension of active colloids is a bacterial suspension. There is a broad understanding in the dynamic effects and pattern formation due to the interaction of bacteria among themselves and the medium. Some studies deal with how environmental changes affect bacteria trajectories, how flagellar motors work and their effect on propulsion^8–12^. Others analyse hydrodynamic interactions between bacteria and surfaces and how confinement modifies their behaviour^13–17^. In all of them, the main interest is the analysis of free swimming cells. However, in most ecological niches, bacteria are found in communities where they may present peculiar density-dependent collective effects^18–22^. Indeed, it has been shown that suspensions of swimming bacteria exhibit contrasting characteristics from those of individual cells, such as long orientation and velocity correlation lengths, cluster instabilities like vortexes, density fluctuations^23^ and bacterial turbulence^13^. Nevertheless, the emergence of collective motion in bacteria suspensions is typically analysed under physical constrains like agar plates or microfluidic devices, where the surface-cell interaction is always present^24–28^. Simulations and theoretical studies have considered configurations without surface interaction^29–33^, and experimental approaches under these conditions study contact-less confinement of λ-DNA molecules and *E.coli* and *Listeria* bacteria using optical tweezers^34,35^ and the formation of flow-induced bacterial streamers in microfluidic flow systems^27^.

Here, we report on the confinement of bacteria in an acoustic levitating environment. The technique involves the modification, by external acoustic forces, of the direction of the bacteria displacement, forcing a confinement in density energy hot spots^36–38^. In order to characterise aggregation processes, typical applications of acoustic traps in inert systems include the trapping of latex beads in different media^39,40^. In the work of Takatori *et al*.^4^, for example, acoustic trapping of Janus particles was used to measure the unique mechanical pressure exerted by self-propelled bodies, i.e. swim pressure. Also, acoustic traps have enabled the label-free monitoring for bio-assay, viability assays and bead-based chemical synthesis, the 3D contact-less positioning of small cell populations and the detection of bacteria in blood samples^41^.

The configuration of our acoustic trap allows us to have a dense population of motile cells in levitation at the nodal plane, avoiding surface interactions. In addition, the confinement of bacteria at this region gives rise to steady floating clusters that do not need a seed to nucleate, as in the case of streamers that typically require a wall-attached biofilm. Altogether, we show that our experiments are useful to elucidate some of the phenomena arising in bacteria societies: such as clustering or aggregation and collective motion^18–28^.

The possibility that bacterial levitating clusters induce the formation of aggregates or artificial biofilms at the nodal plane of the acoustic field is a compelling prospect. This paper does not claim that this possibility has been demonstrated yet, but it reports the first results indicating that enduring floating clusters of bacteria are plausible.

## Results

**Figure 1:**
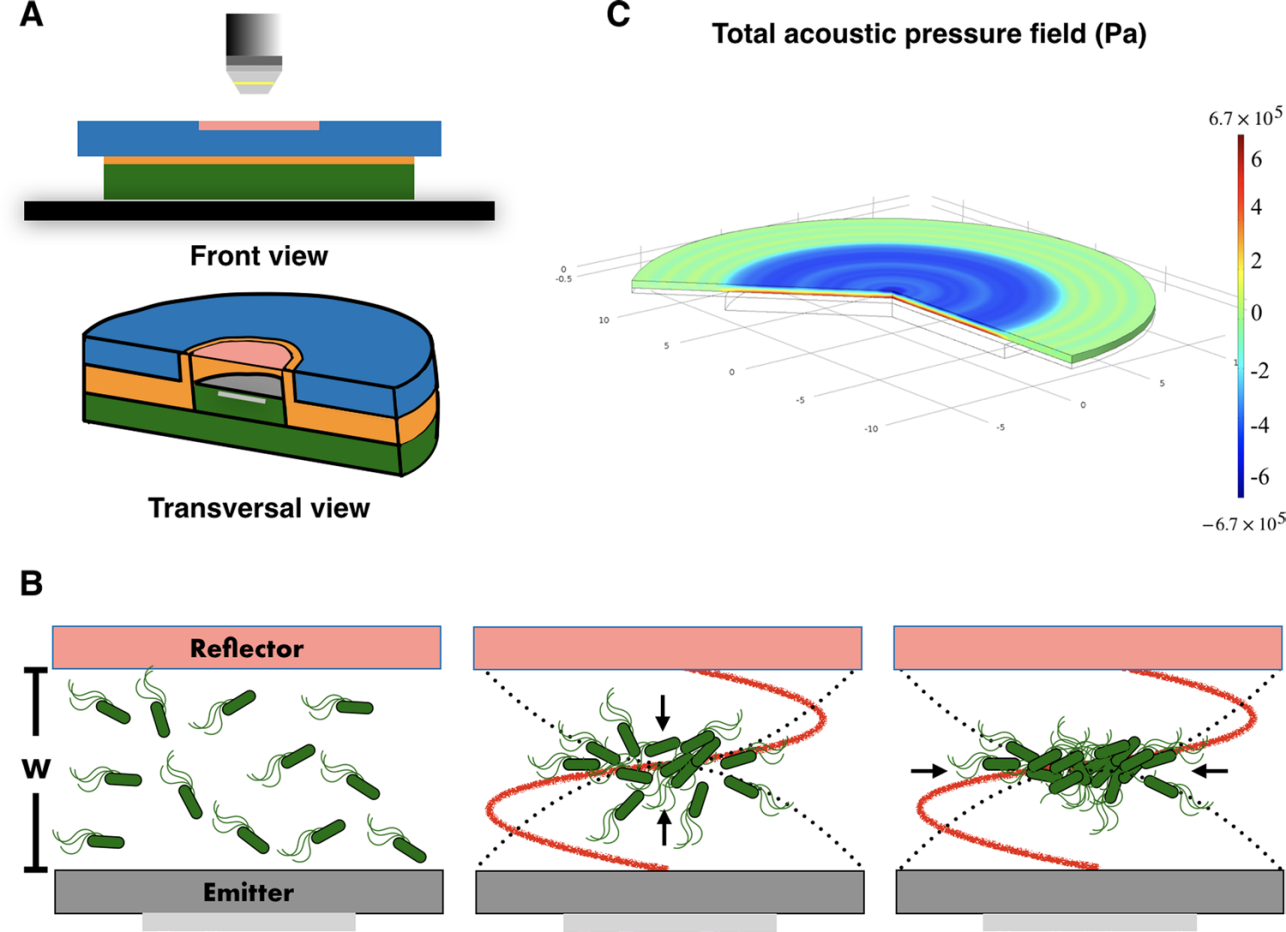
Experimental set up. **(A**) The experimental set up composed by a waveform signal generator (not shown), working in the continuous mode, a signal amplifier (not shown), the acoustic resonator, and an imaging system. As the imaging system, we used a reflection optical microscope assembled with a Hamamatsu camera to record the experiments. The transversal view of the acoustic resonator shows the basic components. The emission plate, where a piezo ceramic transducer is glued to a circular Si wafer (grey), the Mylar spacer (orange) that allows us to modify the emitter-reflector distance, and thereby the number of nodes and anti-nodes in the standing wave inside the pool, and finally, the quartz reflector plate (pink) that is permanently attached to an acrylic cap (blue). **(B)** Details of the pool for bacterial samples. The emitter (grey) and the reflector (pink) plates are separated by a distance w. This follows the relation *w* = *n*(*λ*/2) where λ is the wavelength of the acoustic signal and *n* the number of nodes. The bacterial sample is poured in the pool and the resonator is completely sealed. Soon, the bacteria distribute in all the volume. Once the acoustic field creates a standing wave (red profile), the primary acoustic radiation force (PRF) promotes the bacteria displacement towards the nodal plane. The dotted black line depicts the pressure profile of the wave. The bacteria at the nodal plane are very close to each other due to the presence of the PRF. Then the inter-particle force, also known as the secondary force, enhances the clustering of the cells^36–38^. **(C)** The acoustic pressure field distribution computed by COMSOL simulations. The pressure varies from 6.7 × 10^5^ to −6.7 × 10^5^ Pa, depending on the frequency. In this case (one node) the maximum is at the center of the emission plate. The units of the x,y axis are mm. The color bar (from blue to red) depicts the pressure values above and below the nodal plane.

To confine the *E. coli* cells we designed and built an ultrasonic standing wave trap known as parallel plate resonator. The three basic components are (see, Fig. 1): the emission plate, where a piezo-ceramic transducer with nominal resonance of 2 MHz is glued to a circular silicon wafer with a 5 cm^2^ area and 200 µm thickness. The plate is firmly fixed in a cylindrical support made of stainless steel. Next, there is a Mylar spacer, whose thickness must be half the wavelength of sound in water at the resonant frequency, which for our experiments was 370 µm. The spacer creates a pool between the emission and reflection plates, where the bacteria sample is poured with a pipette before being completely sealed. Finally, the acoustic reflection quartz plate 3λ/4 thick (500 µm) is attached permanently to an acrylic cap, allowing optical access to the chamber. The complete set up comprises the signal generator, amplifier (20X) and the imaging system. The first two supplied the sinusoidal voltage to the transducer. The best driving frequency was 2.1 MHz. Finally, the imaging system is a microscope coupled with a Hamamatsu camera to record the experiments.

COMSOL (Multiphysics® Modeling Software) simulations to model axial radiation forces have been performed, see Fig. 1C. The simulations depict the average energy distribution over the emission plate that corresponds to a circular silicon wafer where the transducer, with a smaller diameter, is attached. The image shows a higher energy zone in the center (blue), where species can concentrate. In real resonators several analogue zones of low and high energy occur, leading to the possibility of several clusters. The simulation visualizes the non-homogenous acoustic energy density distribution. Let us note that in this simulation only transversal excitations have been considered, but other surface waves (Rayleigh, Lamb) propagate through the wafer supporting the transducer, making it difficult to determine the actual energy distribution. What is important for our purposes is the average energy stored in the resonator, which represents the average pressure in the resonator.

### Clustering

In our experiments, a clustering process entails a competition between the pressure induced by the acoustic radiation force, that confines cells into a quasi-2D levitation zone, and the swim pressure of bacteria. It is important to remark that the acoustic field forces the cells together, and, as we discuss later, they spread non-diffusively when the field is removed. This scenario is different when compared to experiments and simulations where rafts of passive particles in an ocean of active particles are observed^30^, or where motility-induced phase separation (MIPS) in diluted suspensions of self-propelled particles occurs^31,32^.

Before getting into details about this clustering process, it is important to emphasize the difficulties to record the complete evolution from the beginning. Firstly, the energy distribution in the trap is not uniform. Therefore, it is likely that several bacteria clusters form in zones where the local energy density is high. Moreover, the system is sensitive to the slightest modification in the parallel plates and this will have an impact in the acoustic energy distribution and therefore, in the position and numbers of clusters.

**Figure 2:**
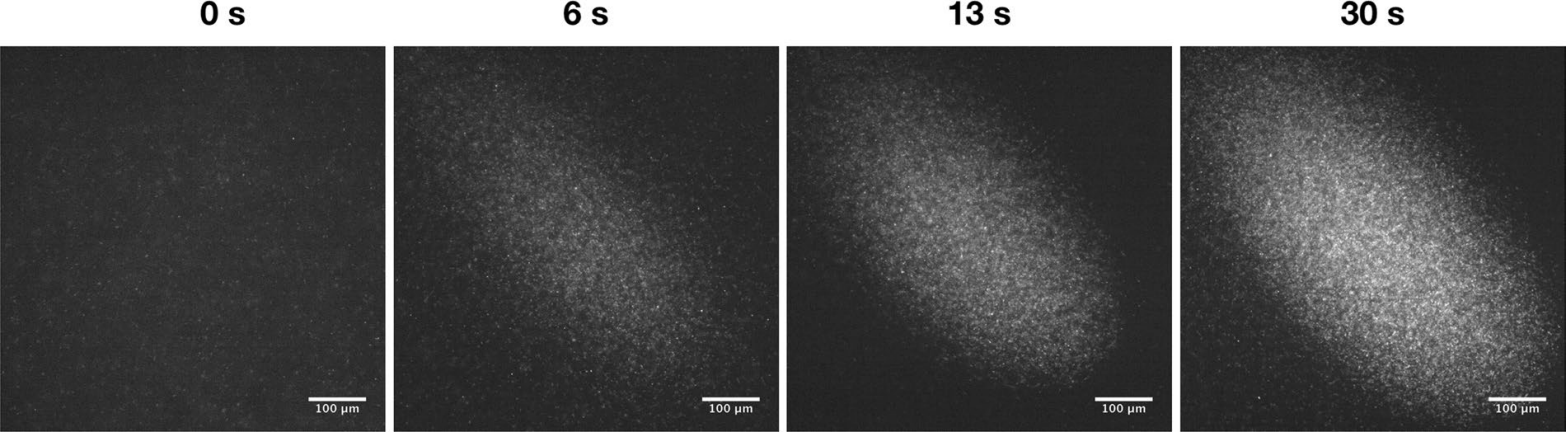
Bacterial clustering in the acoustic trap. Photographs of the induced clustering in the acoustic levitation trap. The pictures were taken at different times; the first one corresponding to the free suspension and the following 6, 13, 30 s after the acoustic field interacts with the bacterial cells inside the acoustic trap.

In Fig. 2, we present a representative experiment of the complete clustering process. For all the experiments *Escherichia coli* RP437-pZA 3R-YFP cells were grown in a minimal medium and re-suspended in a motility buffer (see Methods for further details). This buffer has the right amount of nutrients for the bacteria to be motile, but prevent cellular division^42^, conserving throughout the experiment the bacterial concentration of the sample. In the cavity of the acoustic trap, a sample of the inoculated suspension was deposited and confined between the two plates. At the beginning of the experiment, before the acoustic input, the bacteria cells are distributed in the entire volume of the cavity, as we can see in the first photograph of Fig. 2. Here the bacteria perform their typical reorientation behaviour of run and tumble^8^, constantly modifying their swimming trajectories. Afterwards the sample was exposed to the acoustic field, where the emission plate was excited with a continuous sinusoidal signal at 2.1 MHz and 20 Vpp. The field promotes the bacteria displacement towards the nodal plane followed by a focusing and confinement of the sample (see the photographs at 6, 13 and 30 seconds). Movies were recorded at 30 frames/s (see for example Supplementary Movie 1).

The clustering process can be described by following the area fraction occupied by bacteria inside

concentric rings at each temporal step. This quantity is given by the following expression:

1 $$g(r)=\frac{[(M(r+{\rm{\Delta }})-M(r))]}{\pi [{(r+{\rm{\Delta }})}^{2}-{(r)}^{2}]},$$

where M is the dimensionless mass (intensity of the nonzero pixels) normalised by the total area of a ring with radius *r* and thickness Δ. This concentration profile was obtained following the procedure described in the Methods section.

**Figure 3:**
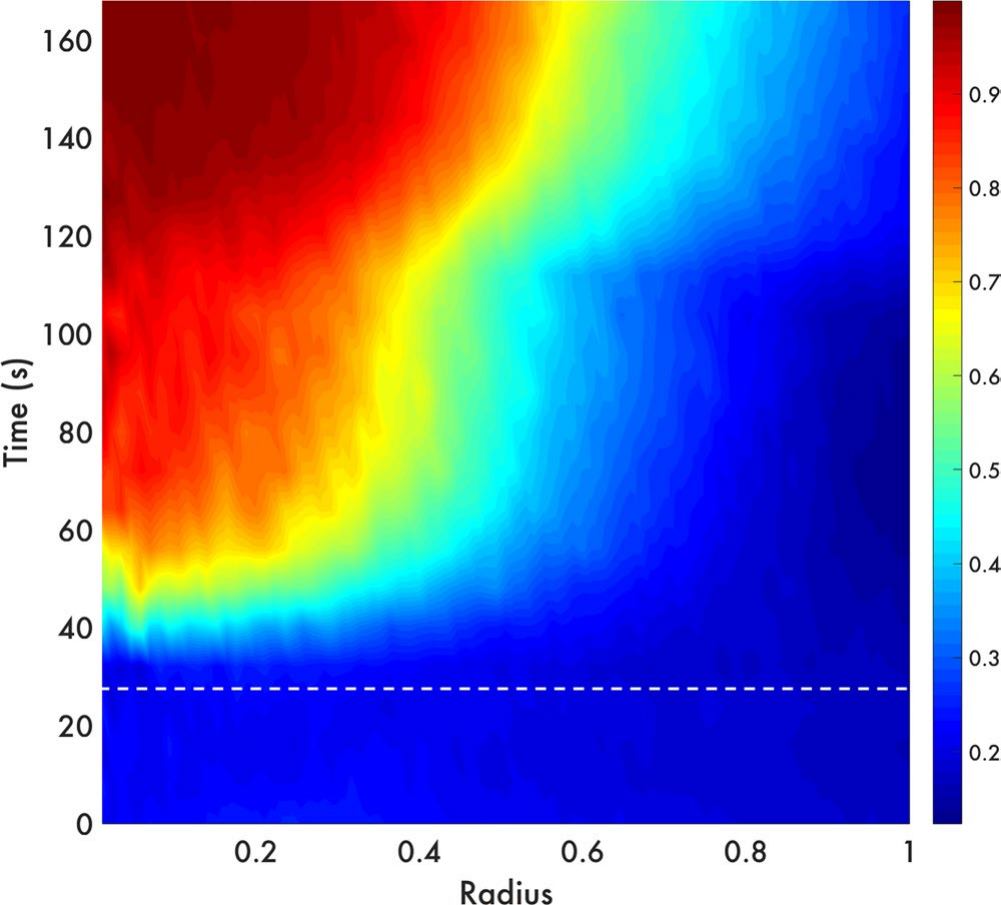
Spatiotemporal distribution of the area fraction in the clustering process. The bacterial concentration increases in the centre of the region once the acoustic trapping begins (indicated by the white dotted line). It takes 20 s to form a highly dense aggregate from an initial homogenous distribution. When the cluster is stable, in other words, when there is no input of bacteria towards the center, we can distinguish three regions. As we can see in the image: the red region with the highest concentration, the yellow-blue part with an intermediate concentration, and the dark blue with a gas-like phase, where bacteria cells are still free to move in their run and tumble fashion. The color bar (from blue to red) depicts the increasing values of area fraction.

As we can observe in Fig. 3 and Supplementary Movie 1, the bacteria cells are driven by the primary radiation force towards the levitation plane at 185 microns above the surface of the emission plate in the first seconds after the acoustic input is applied (t = 30 s). During this clustering process, the fluid is dragged and a back flow is immediately established in the cavity, triggering an advective flow that enables the competition between the bacteria activity and the transversal and axial components of the acoustic radiation force. This generates a perturbation in the initial concentration profile similar to the perturbation observed in a system of highly active colloids (Janus particles)^15^. Thereafter, bacteria get close to each other prompting cell to cell interactions inside spherical or spheroidal clusters that take approximately two and a half minutes to consolidate (see Fig. 3 and Supplementary Movie 1). It is important to remark that ellipsoidal clusters form due to small imperfections in the acoustic resonator (for example, misalignment), that produce inhomogeneities in the pressure at the nodal plane.

Figure 3 shows the concentration profile of bacteria resulting from the pressure gradient. At the centre of the profile (r = 0) we observe a denser accumulation, which comprises 87% of the cluster. Here the bacteria are jammed and impeded to freely swim. Bacteria in these conditions cannot migrate towards the exterior of the confined region. Away from the centre, the profile drops due to the reduced magnitude of the acoustic forces, allowing bacteria to displace in the medium but still unable to escape from the trap.

In Supplementary Movie 1 a recirculation phenomenon is observed, similar to the one reported by Nash *et al*.^26^. This is an indication of a collective or swarming-like motion. We would like to remark that our acoustic technique allows us to control bacteria accumulation inducing collective effects. Among these effects, we found that when bacteria are very close (in the nucleus of the cluster as shown in Fig. 3) an entangled cluster arises. We will discuss this at the end of the next section.

### Spreading

**Figure 4:**
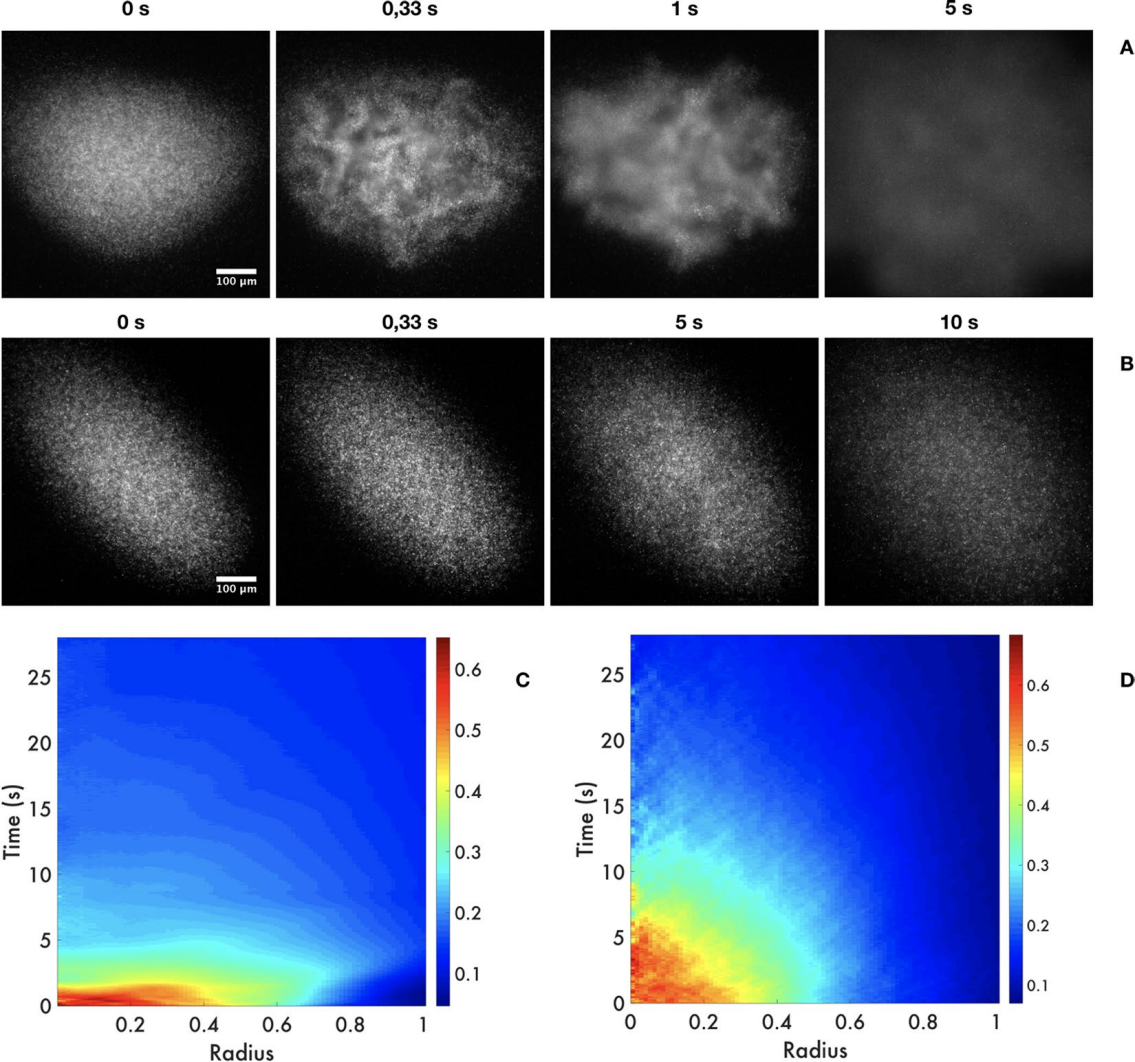
Spreading of bacterial clusters in levitation. In the top panels (A,B) we depict sequences of two representative experiments during the spreading of bacterial clusters. We show four photographs, from the situation where the bacterial communities are completely confined to the case where they are fully dispersed. We show in (**C,D**) the spatiotemporal area fraction. In both cases time zero corresponds to the final cluster and then, the spreading progress with time. The color bar (from blue to red) depicts the increasing values of area fraction.

Our experiments regarding bacterial spreading started with stable bacterial bundles confined by the acoustic radiation force at the nodal plane (185 µm over the reflector surface), as depicted in the first photographs of Fig. 4 (at 0 s). As soon as the acoustic forces cease, the bacteria are free to escape as shown in the subsequent photographs. In other words, the withdrawing of the acoustic forces liberates bacteria and spreading occurs. In Supplementary Movie 2, we notice how the highly concentrated 2D cluster expands after a swarming-like motion: a dilution of the cluster takes place, leaving bacteria in and out of focus. This means that the 2D levitating bacteria community diffuses either horizontally or out of plane towards the walls of the resonator, as they normally do in the absence of acoustic forces^2^. As in Fig. 3, the lower panels of Fig. 4 depict the spatiotemporal evolution of bacteria population as they disperse when the acoustic field is withdrawn. The first cluster (see upper panel) disintegrates in about 5 seconds; the second one in 10 seconds. In Supplementary Movie 3 we see this time a larger ellipsoidal cluster, that once the acoustic field is shut down spreads smoothly. We believe that the difference in the behaviour in both representative experiments in Fig. 4 is due to a difference in the total concentration of the bacteria within the aggregate (note that the spherical cluster is denser. This may be because the cluster is within a higher energy spot).

**Figure 5:**
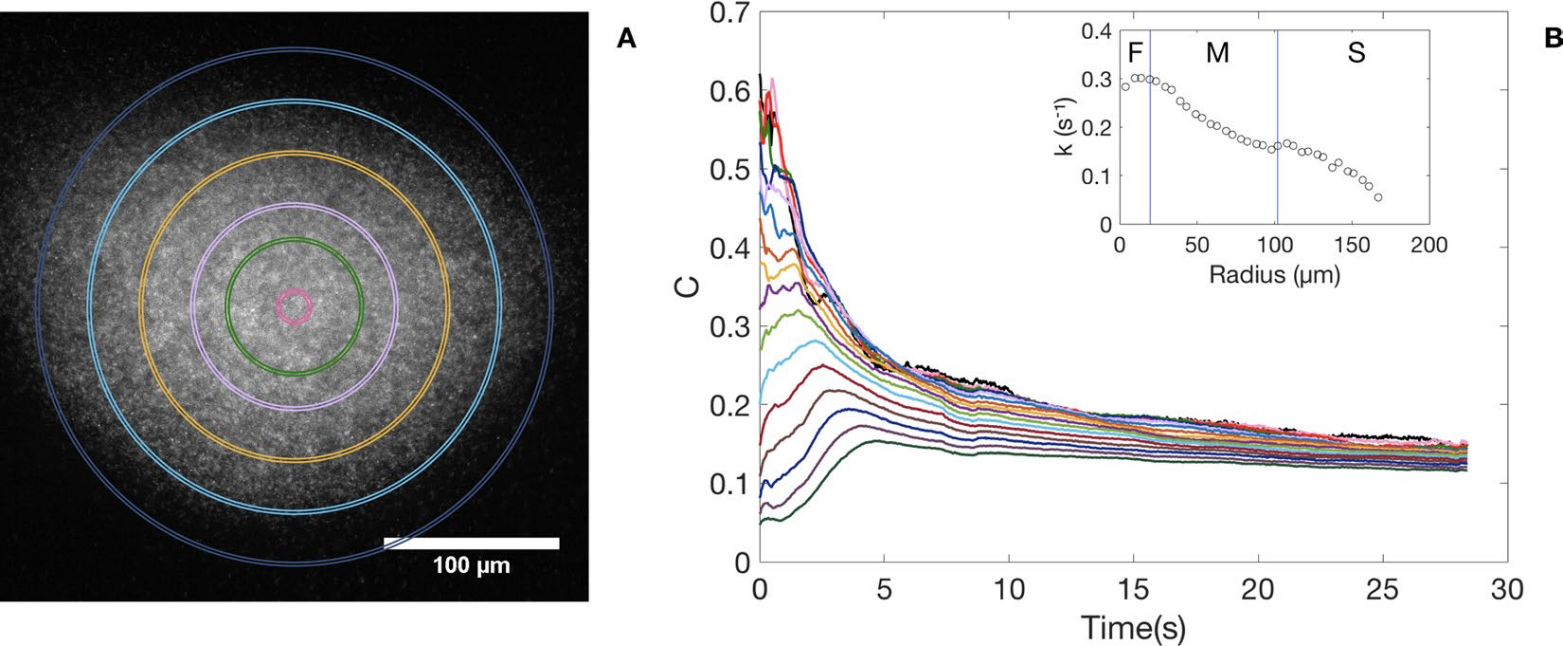
Bacteria concentration decays as a function of r. (**A**) The bacterial cluster is divided in 16 concentric rings of thickness q (for clarity, only 5 are shown). In each one of them we estimate the bacteria concentration in terms of the intensity in grey scale normalised by the area of the ring. (**B**) The concentration as a function of time. Top-to-bottom curves correspond to inner-to-outer rings. Note that the decays are exponential, where in the inner rings the decaying is faster. Indeed, the decays at the external zones of the bacteria cluster are almost constant. In the inset the decay constant for each ring as a function of the ring radius. We see three different behaviours with fast, medium and slow decaying rates.

We now focus on the bacterial spreading dynamics as a function of time, see Fig. 5. The division of the entire cluster in thin rings allows us to follow the dynamics at different radii of the spreading process, clearly observing a transient marked by the inflow and outflow ring by ring. This behaviour persists for any cluster, although the transient reduces when they are more circular.

To describe the spreading kinetics of the bacteria cluster in each ring let us denote *C* as the concentration within a given area and time. The initial value *C*_0_ corresponds to the bacteria being acoustically trapped at a time *t*_0_ = 0 and a final concentration *C*_*f*_ when bacteria are fully dispersed in the complete area at *t*_*f*_  =  ∞. We assume that the spreading is a first order process with a rate proportional to (C − *C*_*f*_):

2 $$\frac{dC}{dt}=-k(C-{C}_{f}).$$

The solution of equation 2 is:

3 $$C=({C}_{0}-{C}_{f}){e}^{-kt}+{C}_{f},$$

which means that *C* reduces exponentially with time. Firstly, note that all curves converge approximately to *C*_*f*_, indicating that bacteria spread homogeneously. Secondly, for the last five rings *C*_0_ − *C*_*f*_ is negative, and this implies that the rings have empty segments due to the elliptical geometry of the aggregate. It takes less than 5 s for these rings to be replenished by bacteria from the inside. Thereafter, the decay is exponential. The values of k, which are plotted in the inset of Fig. 5B, clearly reveal three decaying behaviours: constant with a fast decaying rate (F), concavely decreasing with a medium rate (M) and convexly decreasing with a slower rate (S). Assuming the average values for these regions as 0.3, 0.2, and 0.1 s^−1^, respectively, we can estimate the characteristic spreading speeds, *s* = *qk* (the product of the ring thickness and decaying rates) to be 1.2, 0.78 and 0.39 µm/s.

## Discussion

The magnitude of the vertical acoustic forces, measured experimentally in our trap, are in the order of piconewtons (data not published). Even though we do not measure the transversal forces, it is well known that they are two orders of magnitude smaller than primary radiation forces^36^. Both confine motile bacteria cells at the nodal plane in thin disk-shaped clusters, restricting their degrees of freedom. We observe that the dynamics of the levitating bacteria resembles a self-assembled pump-like structure, comparable to the structure predicted by Nash *et al*.^29^ in their simulations using a lattice Boltzmann in harmonic traps, where they associate the origin of the instabilities to a symmetry-breaking swarm of swimmers.

Our experiments begin with a diluted suspension where, during the elapsed time before the acoustic field is turned on, MIPS^31,32^ is not observed. However, when the acoustic field is activated, the local density suddenly increases at energetic hot spots in the nodal plane, triggering the emergence of clusters and collective effects. In long acoustic confinements, a phase separation may occur (see Supplementary Movie 4): Bacteria inside the clusters, that are not as motile as before, entangle, whereas free (and faster) bacteria remain swimming at the periphery of the clusters.

Although the question of how the phenotype of bacteria (different motility, i.e. motile and nonmotile bacteria, hyper motile bacteria) affects the emergence of collective motion is an appealing question that is actively investigated^29,30^, we have noticed that when bacteria cells are fast swimmers (26–30 µ/s) the acoustic confinement induces a collective behaviour, but when they are slow (10 µ/s, close to the lag phase) this behaviour is not displayed.

If the acoustic trap is suppressed after a few minutes, the floating confined cluster diffuses back into the bulk following an exponential decay, see Fig. 5. The cells are free to escape and they spread around the volume. However, we would like to comment on an important point. When the acoustic field is turned off after a much larger time, the bacterial cluster spreads more slowly. This suggests that bacteria may entangle inside the cluster and experience a reduced ability to swim away from the place where they were confined. In long experiments (approximately 3 hours), the denser region of the cluster does not disperse when the field is removed (see Supplementary Movie 4, where we can see that after a fast sedimentation it is again recovered when the field is turned on). Therefore, it seems that bacteria suffer a strong entanglement with a concomitant aggregation. Similarly, when bacteria are attracted to surfaces^21–28^, they also concentrate and lose motility, eventually forming biofilms. Biofilms aggregate together via complex interactions involving proteins, DNA, intercellular signaling, flagella, and pilli^18^.

Since we still need to perform biochemical analysis of the clustered bacteria, looking for the mentioned signals, it is too soon to claim that the stable clusters we observe in our long- confinement experiments are bacterial aggregates. Nevertheless, from a physical point of view, the first condition to create a biofilm has been attained: bacteria become crowded at a specific place and complex interactions modify their collective behaviour. In our case, this process was enhanced by an acoustic field where the surface interaction can be neglected and where there is no need of surface-attached biofilms or aggregates as seeds. This will give us crucial knowledge of biofilm development without surface interaction, possibly to produce models to evaluate antibiotics in a rapid and cheaper way. Although *Escherichia coli* is not the typical example of biofilm-former bacteria, is a relevant model organism for the study of surface colonisation^22,23^.

In conclusion, we have performed acoustic confinement experiments were *Escherichia coli* cells cluster or aggregate in microgravity-like conditions.

## Methods

### Bacteria culture

Escherichia coli RP437-pZA3R-YFP was cultured overnight in M9 minimal media supplemented with casaminoacids at 30 °C and aerated with orbital shaking at 250 rpm until they reached an optical density of 0.5 (600 nm). At this point bacteria have a mean velocity of 25 μm/s and the suspension a cell concentration around 10^8^ CFU/ml. The overnight suspension was centrifuged for 5 minutes at 5000 rpm and a pellet was obtained. Next the supernatant was discarded and the pellet resuspended in a motility buffer supplemented with D-L Serine that inhibits cellular division. Subsequently, we made a dilution of the sample to have a working suspension with a fixed optical density of 0.1. The strain that we picked (RP437) is a wild type strain for chemotaxis.

To perform the experiments, we pour 250 µl of the inoculated suspension in the pool of the acoustic resonator; thereafter we put the reflector plate on top and removed the excess of the suspension. Prior to any acoustic confinement experiment we tracked the trajectories of the bacteria using Fiji’s Track mate to ensure that the velocity of the bacteria was around 24–26 µm/s for all the experiments. Immediately afterwards, we excite the emission plate with a continuous sinusoidal signal with a driving frequency of 2.1 MHz with 20 Vpp. We manually search for the area were the energy density is higher and the clustering process occurs, then the videos were recorded at 30 f/s with a Hamamatsu camera.

### Image analysis and area fraction distribution

Movies were recorded using an Hamamatsu Orca-Flash 4.0 camera with the software HCI-Image Live. The recording settings were fixed with a Binning 1, the size of the image as 1024 × 1024 Pixels and a depth of 16 bits. The exposure time was 30 ms. Using a custom-made Matlab code, the grey scale images were improved using a median filter that replaced each pixel with the median value in its 3 × 3 neighbourhood. This reduced the background noise. Later we performed a background correction, where we considered the first frame, corresponding to the nodal plane where no bacteria are in focus, as the background and subtracted it from the following frames. To analyse the intensity variations as a function of time, we created a circular mask positioned at the center of the image. The mask grows radially with a step corresponding to the average length of a bacteria. This allows us to obtain a pixel intensity within 85 rings per image. The rings cover the complete image area.

We used the followed intensity criterion: the number of what we considered is the background are those pixels with an intensity between 0 and 350, and bacteria those from 351 to 65535.

Finally, we normalise the number of «bacteria pixels» with the total area of the ring to obtain the area fraction. We evaluate all the frames to get a spatiotemporal distribution of the area fraction. To perform this analysis we assume that due to the contrasting magnitudes of both forces (vertical and transversal), the floating cluster has a disk shape whose thickness is negligible and can be considered as 2 dimensional.

## Electronic supplementary material

Movie 1

Movie 2

Movie 3

Movie 4
